# Genome-Wide Identification of Putative MicroRNAs in Cassava (*Manihot esculenta* Crantz) and Their Functional Landscape in Cellular Regulation

**DOI:** 10.1155/2019/2019846

**Published:** 2019-06-19

**Authors:** Amika Yawichai, Saowalak Kalapanulak, Chinae Thammarongtham, Treenut Saithong

**Affiliations:** ^1^Systems Biology and Bioinformatics Research Group, Pilot Plant Development and Training Institute, King Mongkut's University of Technology Thonburi (Bang Khun Thian), Bangkok, Thailand; ^2^Bioinformatics and Systems Biology Program, School of Bioresources and Technology, King Mongkut's University of Technology Thonburi (Bang Khun Thian), Bangkok, Thailand; ^3^Biochemical Engineering and Pilot Plant Research and Development Unit, National Center for Genetic Engineering and Biotechnology at King Mongkut's University of Technology Thonburi (Bang Khun Thian), Bangkok, Thailand

## Abstract

MicroRNAs are small noncoding RNAs, involved in the regulation of many cellular processes in plants. Hundreds of miRNAs have been identified in cassava by various techniques, yet these identifications were constrained by a lack of miRNA templates and the narrow range of conditions in transcriptome study. In this research, we conducted genome-wide analysis identification, whereby miRNAs from cassava genome were thoroughly screened using bioinformatics approach independent of predefined templates and studied conditions. Our work provided a catalog of putative mature miRNAs and explored the landscape of miRNAome in cassava. These putative miRNAs were validated using statistical analysis as well as available cassava expression data. We showed that the crowded locations of cassava miRNAs are consistent with other plants and animals and hypothesized to have the same evolutionary origin. At least 10 conserved miRNAs were identified in cassava based on the comparative study of miRNA conservation. Finally, investigation of miRNAs and target gene relationships enabled us to envisage the complexities of cellular regulatory systems modulated at posttranscriptional level.

## 1. Introduction

MicroRNAs (miRNAs) are endogenous noncoding RNAs (ncRNAs) that are approximately 18-25 nucleotides (nt) in length. To express their regulatory function, miRNAs must undergo series of steps as described in their biogenesis [1]. MicroRNAs are encoded in the genome as long primary transcripts called primary miRNA (pri-miRNA), when they are expressed from MIR genes, under the regulation of RNA polymerase II (RNA pol II) [2]. The pri-miRNA transcripts are processed to precursor miRNAs (pre-miRNAs) by the dicer-like protein 1 (DCL1). The pre-miRNAs are later diced into short miRNA duplexes containing one or two mature miRNAs.

MicroRNAs regulate target genes posttranscriptionally. They bind to the target mRNAs and cause either mRNA degradation or translation inhibition [3, 4]. Through these mechanisms, miRNAs have been reported to play important roles in cellular regulations underlying growth, development, and stress responses. For instance,* lin-4* and* let-7* of* C. elegans* were found to regulate the schedule of larva development [3, 5]. miRNA-124, which controls neural development in vertebrates [5], miRNA-21, miRNA-196, miRNA-17-5p, miRNA-20a, and miRNA-106a play a role in the human immune system [5]. In plants, miR169 family was found to regulate flowering in transgenic Arabidopsis [6] and several miRNAs were involved in the regulation of embryogenesis in maize [7]. The characteristics and regulation of miRNAs in animals and plants were compared in several studies [4, 8]. Although miRNAs genes are transcribed under the regulation of RNA pol II, in both plants and animals and the mechanistic processes and operating compartments differ. In plants, miRNA biogenesis requires DCL1 protein to process pri-miRNAs transcripts to pre-miRNAs, whereas, in animals, the Drosha protein is used. Furthermore, the binding of miRNAs to their targets is more perfectly complemented in plants than in animals, and it is shown that, in plants, the influence of miRNA on cellular regulation is by mRNA degradation rather than translational inhibition as expected in animals.

Since the discovery of the first two miRNAs*, lin-4* and* let-7*, in* C. elegans* [3], great efforts have been made to explore unknown miRNAs and unravel their functions, regarding posttranscriptional regulation. For decades, numerous putative miRNAs, many of which were later found to be miRNA candidates, have been continuously identified and reported in a broad range of organisms, including humans [9] and plants [2, 4]. After decades of the first miRNA discovery, at least 35,828 mature miRNAs from 223 species have been identified (miRBase Release 21 [10]), which have contributed immensely to our knowledge of miRNAs. MicroRNA study in plant species began in 2002 when they were identified and reported in Arabidopsis. To date, miRNA studies have been conducted in many plant species, including rice (*Oryza svita*), soy bean (*Glycine max*), potato (*Solanum tuberosum*), barrel medic (*Medicago truncatula*), maize (*Zea mays*), and cassava (*Manihot esculenta*). The roles of miRNAs in cellular regulation have also been researched along with the identification, such as the roles of miR156 in increasing tiller number in rice [11] and miR169 in regulating stomatal opening and transpiration rate in tomato under drought stress condition [12]. Notwithstanding these research achievements, our knowledge of miRNAs, especially for essential staple crops, is limited.

Cassava is an essential staple crop utilized as a source of food and feed, whose relevance is predicted to increase in the future. In contrast, knowledge of miRNAs in cassava is little and mainly explored by two identification approaches: a homologous-based method [13–15] and a high-throughput RNAseq method [16–20] (Rogans et al., 2016). The homologous-based method is capable of finding cassava miRNAs that are widely conserved with miRNAs from other plants that share common ancestors [13–15]. The RNAseq technique is an expression-based method that speeds up exploration of miRNAs by the high-throughput measurement. This method, moreover, enables us to find the novel miRNAs independent of the prior evidence of their homologs. With the advent of next-generation sequencing (NGS) technology and powerful bioinformatics tools, a number of putative miRNAs have been discovered in cassava [16–20] (Rogans et al., 2016). However, these methods are only able to capture the miRNAs expressed under the investigated conditions such as stress responses [16, 17, 19, 21] and development [18, 20], thereby limiting prospects for new miRNAs discovery. To expand the coverage of genome-wide miRNA investigation, an alternative method was recently proposed by coupling exhaustive search algorithm with an efficient pre-miRNA predictor [22]. HeteroMirPred predictor [23] was selected particularly for the framework as it includes self-containment discriminative feature. It was found that different RNAs classes possessed diverse self-containment index values, so that helps screen miRNAs from the other small RNAs containing similar size as concerned by Axtell and Meyers, 2018 [24]. High self-containment was observed in miRNAs but not in other RNA classes in which they showed much lower self-containment values. This suggested self-containment as a useful feature for enhancing robustness of prediction on miRNA structures [25]. The self-containment index and its derivatives are also exploited in other miRNA prediction tools developed recently [26–28]. The proposed method of Yawichai, however, was only applied for intronic miRNA prediction [22].

To the extent of exploring the entire miRNA members in cassava, this study was carried out to investigate the entire noncoding sequences of the cassava genome, identify putative miRNAs, and explore the landscape of the miRNAome. Using genome-wide approach, we identified a number of putative miRNAs in cassava. The predictions were validated statistically and by use of cassava expression data. Since the employed method is independent of templates from previous identifications and experimental conditions, predicted results can be considered to include nearly all putative miRNAs in the cassava genome. Not only did this study provide the largest resource of putative miRNAs in cassava, but it also contributed to an overview of the landscape of miRNAs and their functional relevance in cellular regulation in cassava. It is one of only few studies that explored miRNA population density in the genome. Moreover, it is the first study in cassava that outlined the global view of posttranscriptional regulation driven by miRNAs at genome level. The molecular functions tightly regulated by miRNAs were discussed. Moreover, miRNAs with multifunctions involved in diverse processes were proposed and suggested to play roles in a coordination of cellular regulation. Finally, we showed that our genome-wide identification captured extensive numbers of miRNAs involved in both storage root development and starch biosynthesis, critical for cassava establishment.

## 2. Materials and Methods

### 2.1. Data Processing

Noncoding sequences were extracted from the cassava genome (*Manihot esculenta *Crantz V4.1 from Phytozome V9 (https://phytozome.jgi.doe.gov). The sequences were separated into two types, based on genomic region, namely, intergenic sequences, located between two genes which precede or follow an individual gene, and intronic sequences, located within a gene between 5' untranslated regions (5'UTRs) and 3'UTRs (Figure S1 (box  I)). In order to prepare input sequences for HeteroMirPred [23], noncoding sequences were chopped to 120 nucleotides (nt) with 20 nt overlap sliding window. For the last sliding window which was shorter than 120 nt, the sequence was collected backward to gain 120-nt length (Figure S1 (box  II)).

### 2.2. MiRNA Identification

The pipeline for miRNA identification was separated into two main parts, pre-miRNA location screening and putative mature miRNA prediction (Figure S1A (box  III)). For the screening process, 120 nt input sequences were submitted to HeteroMirPred. The sequences that passed the criteria of HeterMirPred, herein using highest cut-off score (= 1), were defined as the predicted sequences. The predicted sequences that overlapped in location were merged into one single locus denoted as a predicted locus, to avoid sequence redundancy. Sequences that matched rRNAs and tRNAs were excluded by sequence similarity search using BLAST. Subsequently, the predicted loci were processed for putative miRNA prediction based on their secondary structures folded by Mfold V.3.6. The putative miRNA duplexes (stems of hairpin structures) were ascertained according to the following criteria: length of strands > 15 nt, asymmetric bulge ≤ 2 nt, and mismatch ≤ 4 nt. Then, putative mature miRNAs, defined as single strand RNAs with length > 15 nt, were identified from both strands of putative miRNA duplexes. In the case that miRNA duplexes of the predicted loci could not be identified, sequences of such predicted loci were extended up to 20 nt from both ends and reexamined.

### 2.3. Statistical Analysis

Previously reported cassava pre-miRNAs [15–19] which were identified using various methods were served as reference data omnibus, for evaluating the reliability of the predictions. The pre-miRNAs reported in five studies [15–19] were aligned against the predicted loci through BLASTN, for which the pre-miRNAs were pairwise compared by matching their locations in the reference genome (*Manihot esculenta*: AM560 cultivar). The employed criteria were as follows: (1) sequences of the predicted loci shared the same scaffold with the corresponding known pre-miRNAs, (2) overlapping locations between the compared pre-miRNAs were greater than 60 percent of the observed sequences, and (3) mismatches of the aligned sequences were no greater than 2 nt. The hypergeometric probability (see (1) and (2)) for obtaining *X* or more published pre-miRNAs from randomly selecting 83,178 predicted loci without replacement, from a total of 8,279,392 investigated fragment sequences was calculated to prove the statistical significance of our predicted results.

(1) px≥X=1−px≤X−1

(2) px≤X−1=∑i=0X−1kX−1−iN−kn−X−1+iNn

where *k* is the number of known pre-miRNAs in the reference data omnibus,* X* is the number of the predicted miRNAs loci matched with the available known cassava pre-miRNA,* N* is the number of input noncoding sequences, and* n* is the total number of predicted miRNA loci proposed in this study.

### 2.4. Expression Analysis of the Putative Mature MiRNAs

The available transcriptome data of cassava were employed to support the expression of the putative mature miRNAs proposed in this work. They consisted of two sets of small RNA [16, 20] and five sets of total RNA deep sequencing data [18–21, 29]. For small RNA expression data, the expressed sequences were directly BLAST searched against the putative mature miRNAs, whose expression was noted based upon these criteria: percentage of coverage = 100, sequence mismatches ≤ 3 nt, and number of mapped reads ≥ 10, to provide maximum evidence of putative mature miRNAs though more rigorous criteria could be applied. For total RNA expression data, the expressed sequences and the mature miRNA candidates were aligned on the basis of cassava AM560 reference genome using Stampy v.1.0.20 [30] and SAMtools v.0.1.18 [31]. The expressions of the putative mature miRNAs were defined according to the following criteria: percentage of coverage ≥ 60 and number of mapped small RNA sequence ≥ 10, to provide maximum evidence of putative mature miRNAs though more rigorous criteria could be applied.

### 2.5. Conserved MiRNA Identification

The sequences of mature miRNAs from Viridiplantae were collected from miRBase Release 21 [10] and plant miRNA database (PMRD; http://bioinformatics.cau.edu.cn/PMRD/). The retrieved miRNA sequences were pooled together and redundant entries were removed. Through the sequence alignment, the conserved miRNAs were defined according to the criteria: (1) the aligned sequences of plant mature miRNAs were 100 percent covered; (2) mismatches of aligned sequences, including flanked sequences, did not exceed 3 nt.

### 2.6. Population Density Estimation

Putative miRNA genes are clustered in a nonuniform pattern across the cassava genome. The population density (*D*) was a normalized factor, estimating the crowdedness of the miRNA in a defined region of the genome, as given below.

(3) Population  DensityD;miRNA1GB=No.  of  miRNAsLength  of  the  observed  regionGB

The population density of miRNAs was calculated for both intergenic (denoted as *D*_*ig*_) and genic (denoted as *D*_*g*_) regions of the genome. The* D* factor determined from our putative miRNAs were compared with those computed by miRNAs reported in five publications [15–19] and also from miRNAs of six well-studied organisms, namely, Arabidopsis (*Arabidopsis thaliana*), rice, castor bean (*Ricinus communis*), soy bean (*Glycine max*), human (*Homo sapiens*), and worm (*Caenorhabditis elegans*), in miRBase Release21 [10]. Human (GRCh38.p5) and worm genomes and annotation were retrieved from NCBI database (https://www.ncbi.nlm.nih.gov). All plant genomes and annotation were collected from Phytozome database V11 (https://phytozome.jgi.doe.gov).

For a genome, the length of the genic region was calculated from the summation of nucleotide bases of all protein-coding genes, which was then subtracted from the whole genome sequence to estimate the intergenic region.

### 2.7. Gene Target Prediction

The putative mature miRNAs were searched for their target transcripts in cassava genome (*Manihot esculenta *Crantz V.4.1; Phytozome V.9) using two target predictors, psRNATarget [32] and Targetfinder [33], with default setting. Only the consistent predictions were reported and included in further analyses.

## 3. Results

### 3.1. Genome-Wide Identification of MiRNAs in Cassava

The global identification of miRNAs was conducted according to the search pipeline of Yawichai et al. [22]. The method combined sequence analysis techniques with a pre-miRNA predictor, named HeteroMirPred, which has a great performance for miRNA prediction because of the well differentiation between miRNA and siRNA [23]. Firstly, non-protein-coding regions consisting of intronic and intergenic sequences (covering about 98 percent of the whole genome sequence) were extracted from cassava genomic sequence and fragmented into 120 nt subjective window size with 20 nt overlapping. It yielded 8,279,392 fragments which were passed to the HeteroMirPred predictor, where the properties of the 120 nt sequences were determined according to the ubiquitous characteristics of miRNAs. The qualified sequences, containing at least one miRNA, were subsequently defined as the predicted loci (see Materials and Methods, Figure S1A, Table 1). Figure S1 demonstrates the prediction framework and also illustrates the terms of predicted sequences employed in this study. From the overall noncoding fragments, 83,178 predicted loci (~ 2.35 percent of the whole genome sequences) were proposed based on HeteroMirPred criteria, with the highest stringency (Figure S1A). Overlapping fragments were assembled and merged; hence, 65,396 loci were predicted based on secondary structure features via Mfold (Figure S1A). Putative mature miRNAs were inferred based upon the stem-loop arrangement of their secondary structures as exemplified in Figure S1B. To further find the location of the mature miRNAs, we assumed that both arms of the stem-loop structures could accommodate one or more mature miRNAs, according to the criteria (length of strands > 15 nt, asymmetric bulge ≤ 2 nt and mismatch ≤ 4 nt) suggested by Meyers et al. [34] and Thakur et al. [35] (Figure S1B, gray box). Consequently, 153,794 sequences were presumed to have the potential for encoding mature miRNAs (Figure S1A, Table 1).

### 3.2. Statistical Analysis of MiRNA Prediction

Through genome-wide analysis, 83,178 predicted loci, corresponding to 152,111 putative mature miRNAs, were proposed in cassava. The prediction represented the exhaustive numbers of the putative miRNAs containing cassava genome. Hypergeometric probability analysis was conducted to demonstrate the significance and reliability of our predicted loci based upon five publications [15–19]. The results showed that our prediction was statistically significant with hypergeometric* p* value ≤ 0.05 against all datasets, except in case of 58 which had relatively less data available (Table 2). The large number of putative miRNAs predicted in this work may be from (1) the thorough investigation of miRNA-located regions in the genome, and (2) the ab initio approach employed herein that enabled us to capture putative miRNAs beyond the constraints on availability of miRNA templates and experimental conditions, alleviating the limitation of previous works.

### 3.3. Expression Analysis of Putative Mature MiRNAs

In addition to the statistical analysis, expression of the putative miRNAs was investigated based upon ~ 101 million expressed sequences gathered from seven datasets of cassava transcriptome [16, 18–21, 29] to support their existence in cassava. Up to 41.76 percent (63,519 candidates) of total predictions showed expression potential according to the minimal expressed depth criteria, and about 9.79 percent (14,892 candidates) were still valid with the expressed depth ≥ 10 (Table S1). By using the common criteria of an expressed sequence in RNAseq measurement (depth ≥ 10), the results supported expression of 14,878 putative mature miRNAs that have never been identified elsewhere, and also the 14 putative mature miRNAs reported as conserved miRNAs with other organisms.

### 3.4. Array of MiRNAs in Cassava Genome

**Figure 1:**
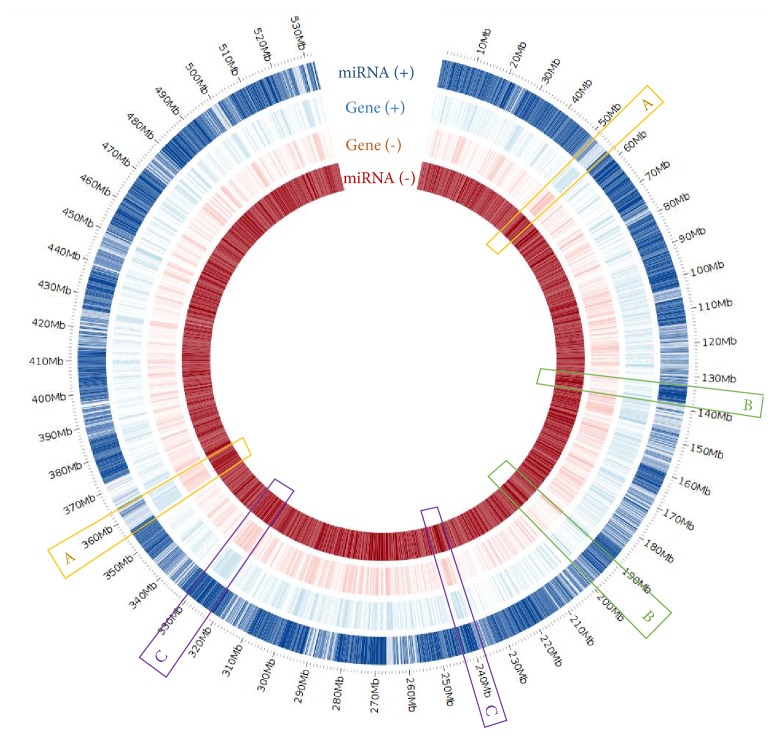
Distribution of miRNAs and protein-coding genes in cassava genome. The “A” frames show that, for regions containing dense genes, miRNAs were sparse. The “B” frames show that, for regions containing sparse genes, miRNAs were dense. The “C” frames show the regions that genes and miRNAs were both dense.

The majority of the putative miRNAs were predicted from the noncoding regions between genes in the genome (Table 1), indicating the similar locations of miRNAs in cassava genomic sequence to that of the other plant species [4, 32, 36].  Figure 1 showed the array of all predicted loci aligned with the locations of protein-coding genes in cassava genome. There was a high frequency of the putative miRNAs in the intergenic regions in the cassava genome. The miRNAs were relatively sparse in the genic regions, in which the miRNA density was opposite to the density of protein-coding genes (e.g., marked as “A” in Figure 1), whereas the reverse pattern was observed in the intergenic regions (e.g., marked as “B” in Figure 1). However, we also observed some hot-spot regions where both miRNA and protein-coding genes are densely located (e.g., marked as “C” in Figure 1). Since the genome structure, for example, the relative span coverage of genic region in the genome, may vary among species [37], the current usual analysis of miRNAs distribution could be more precise by performing the formal analysis to normalize such a variation. Such results would give the canonical information reflecting the reality of miRNA array.

**Figure 2:**
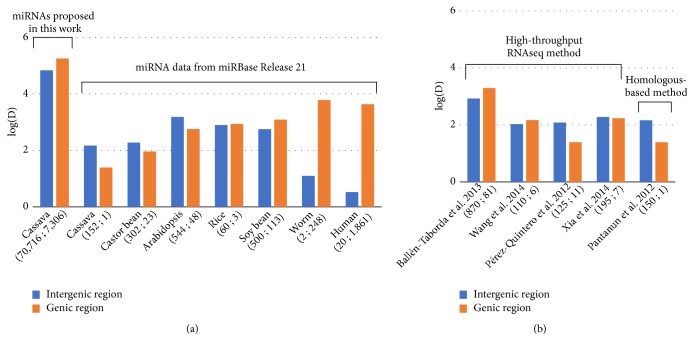
Population density (*D*) of miRNAs in intergenic and genic regions. (a) Population density of the proposed miRNAs in cassava in comparison with that of the other well-known organisms. (b) Population density of miRNAs in cassava derived by the independent sets of miRNA identification studies. The x-axis shows the source of miRNAs. The y-axis shows log of miRNA density in the given regions per one gigabase (GB). The numbers in parentheses show the total number of miRNAs located in intergenic and genic regions, respectively.

To reconcile the bias of genome structure, the pattern of miRNA distribution was theoretically estimated. The population density (*D)* of miRNAs, which refers to the tentative numbers of miRNAs contained in the unit length of sequence, was measured here based on (3) in the Materials and Methods. The results showed that the population density of our predicted loci was higher in genic regions (Figure 2, Table S2). Similar findings have been reported in other well-studied plants with large dataset in miRBase [10] such as rice (*O. Savita*) and soy bean (*G. max*) and in animal species (Figure 2(a)). This study provided a different perspective on the imbalanced locations of cassava miRNAs as well as the arrangement of miRNA in plants. To ensure results were not a consequence of the computationally massive identification, we applied similar analysis to estimate the density of miRNA in cassava genome using various cassava miRNA datasets (Figure 2(b)). Accordingly, it might infer that miRNAs of both plant and animal species came from the same origin, duplication of the protein-coding genes [8, 38], yet the different evolutionary pathways were expected.

### 3.5. Conservation of the Cassava MiRNAs among Plants

**Figure 3:**
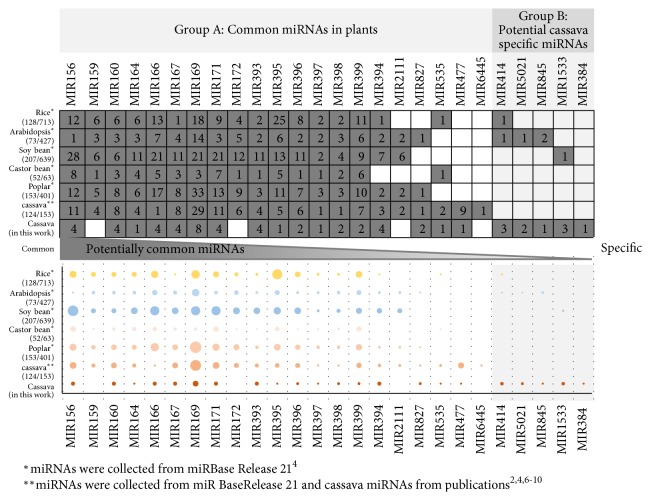
Conservation of cassava miRNAs based upon the miRNAs of organisms in miRBase (Release 21)^10^. The number of miRNAs in each organism classified in 21 miRNAs families is demonstrated in the upper table and the corresponding graph shown below. The numbers in brackets of the upper table represented members of miRNA families as follows: (members of miRNA families conserved between cassava and well-studied plants / number of total miRNA members in each organism). Size of nodes in the below graph denoted number of miRNA members in each miRNA family.

To investigate the conservation of the miRNAs in cassava, 152,111 putative mature miRNA predicted from cassava noncoding sequences were analyzed against the mature miRNAs of plants deposited in miRBase (Release21) [10] and those reported in publications [15–19] (see Materials and Methods). Based on miRNA families found in Viridiplantae, the results showed that cassava contained at least 22 miRNA families, suggested by 59 putative mature miRNAs, conserved with other plants, of which 17 families corresponding to 49 putative mature miRNAs were previously identified in cassava [15–19] (Group A in Figure 3). Interestingly, 5 families of miRNA were at first presented in cassava according to the finding of 10 putative mature miRNAs herein (Group B in Figure 3).

Besides, the functional conservation of miRNA family was observed by comparing the number of miRNA members in a family across plants. The 22 conserved miRNA families of cassava were studied with respect to that of five well-studied plants:* Arabidopsis thaliana *(Arabidopsis),* Oryza sativa *(rice),* Ricinus communis *(castor bean),* Glycine max *(soy bean), and* Populus trichocarpa *(black cottonwood). The results in Figure 3 showed that most of cassava miRNA families in group A were conserved in all studied plants with similar number of members, while the families in group B were only conserved with Arabidopsis, rice, and soy bean (Figure 3). An explanation could be that less conserved miRNA families (Figure 3; group B) might be involved in species-specific regulatory functions, considering that the putative miRNAs in group A were found in many different plants (Figure 3).

### 3.6. Functional Analysis of the MiRNAs in Cassava and Their Roles in the Cellular Regulation System

The miRNAs in cassava have been identified and studied for nearly a decade. While a number of putative miRNAs have been continuously proposed, analysis of roles of miRNAs in cassava was mainly focused on biotic and abiotic stress [16, 17, 19, 21, 39, 40] and also confined by the information of conserved miRNAs in well-studied organisms [15]. According to the genome-wide identification of putative miRNAs in the previous section, the prediction of their gene targets would allow us to envisage the landscape of miRNA function in cassava genome. The 152,111 putative mature miRNAs in cassava were predicted their gene targets. The results revealed the first estimation of overall functions of miRNAs in cassava. In addition, we further investigated the pattern of miRNA-target relationships which would imply miRNA-modulated regulation in cassava.

To observe the overall functions of miRNAs in cassava, the gene targets of the putative mature miRNAs were predicted using two computational tools, psRNATarget [32] and Targetfinder [33] (see Materials and Methods). These tools are practically more effective for predicting targets of the putative miRNAs conserved with known miRNAs; thus, in the case of nonconserved or previously uncharacterized miRNAs proposed in this work, the sequences on both strands of the miRNA duplex stems were employed for their target gene prediction based on complementary sequence. The results showed a large number of genes in cassava genome had potentials to be modulated by the putative mature miRNA. It was found that 25,617 genes (~ 84 percent of genes in genome) were targeted by 23,543 putative mature miRNAs, suggesting tight gene regulation by miRNAs. Corresponding results were also reported in human [41, 42].

**Figure 4:**
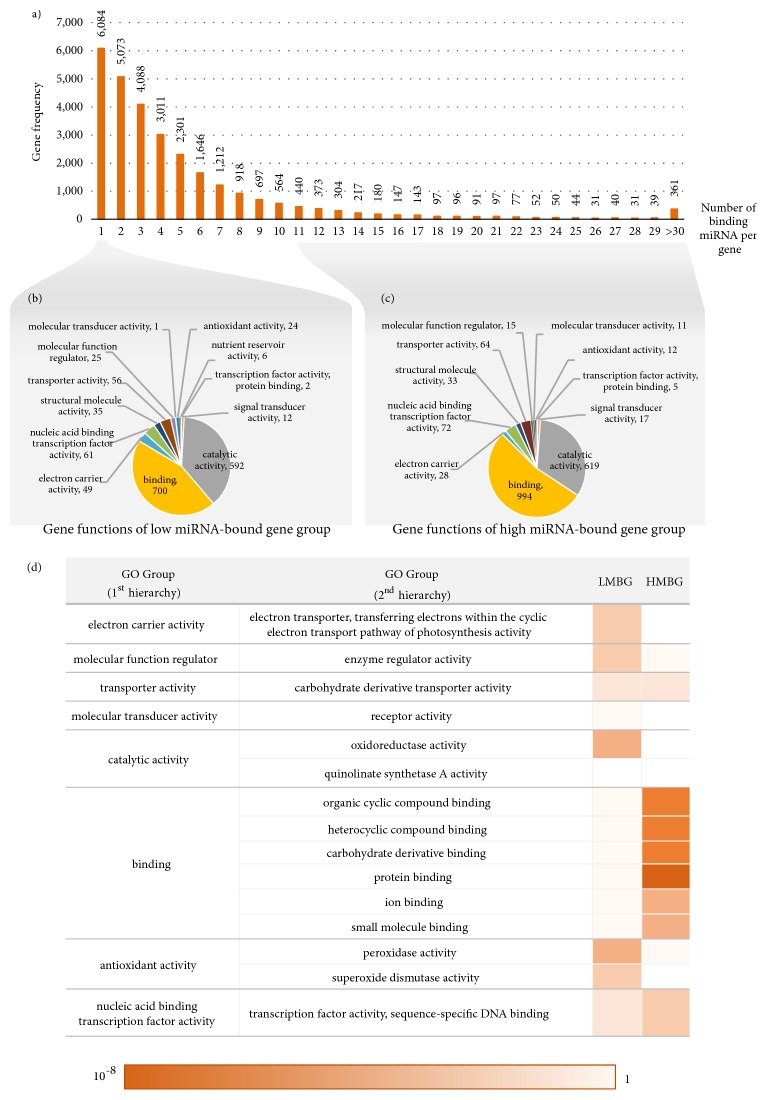
Functional analysis of cassava miRNAs. (a) The distribution of miRNAs-bound genes. (b-c) GO analysis based on molecular function of the selected 10 percentile of miRNA-target genes in low miRNA-bound genes (LMBG) group and high miRNA-bound genes (HMBG) group, respectively. (d) Heatmap represents* p* value of hypergeometric probability for GO enrichment analysis. The enriched functions were proposed when GO terms obtain* p *value < 0.05.

To explore regulatory system modulated by miRNAs, interactions of the putative mature miRNAs and their target genes were analyzed. As a result, 151,087 interactions were proposed between 22,312 putative mature miRNAs and 28,504 target genes. It was found that one putative mature miRNA may interact with up to 197 target genes, whereas one target gene may be bound by one or more miRNA(s), ranging from 1 to 576 miRNAs. The distinct relationships predicted among putative miRNAs were further studied to investigate whether these were benefit cellular regulation. By classifying target genes into two groups based on the number of miRNA-binding sites, low miRNA-bound genes (*LMBG*) and high miRNA-bound genes (*HMBG*) groups were defined by 10th percentile lowest and highest in rank, respectively (Figure 4(a)). About 10 percent of the predicted target genes in each group (2,850 genes) were randomly taken for gene ontology (GO) analysis (Figures 4(b)–4(d)). GO enrichment analysis exhibited the dominant functions of these targets genes which are relevant to binding and enzyme regulator activity (*p *value < 0.05). It also showed that the enriched functions of target genes were different in* LMBG* and* HMBG *groups. The significant functions of genes in* HMBG *group are related to binding activity, while genes in* LMBG *group are more relevant to electron carrier activity, molecular function regulator, catalytic activity, and antioxidant activity (Figure 4(d)).

**Figure 5:**
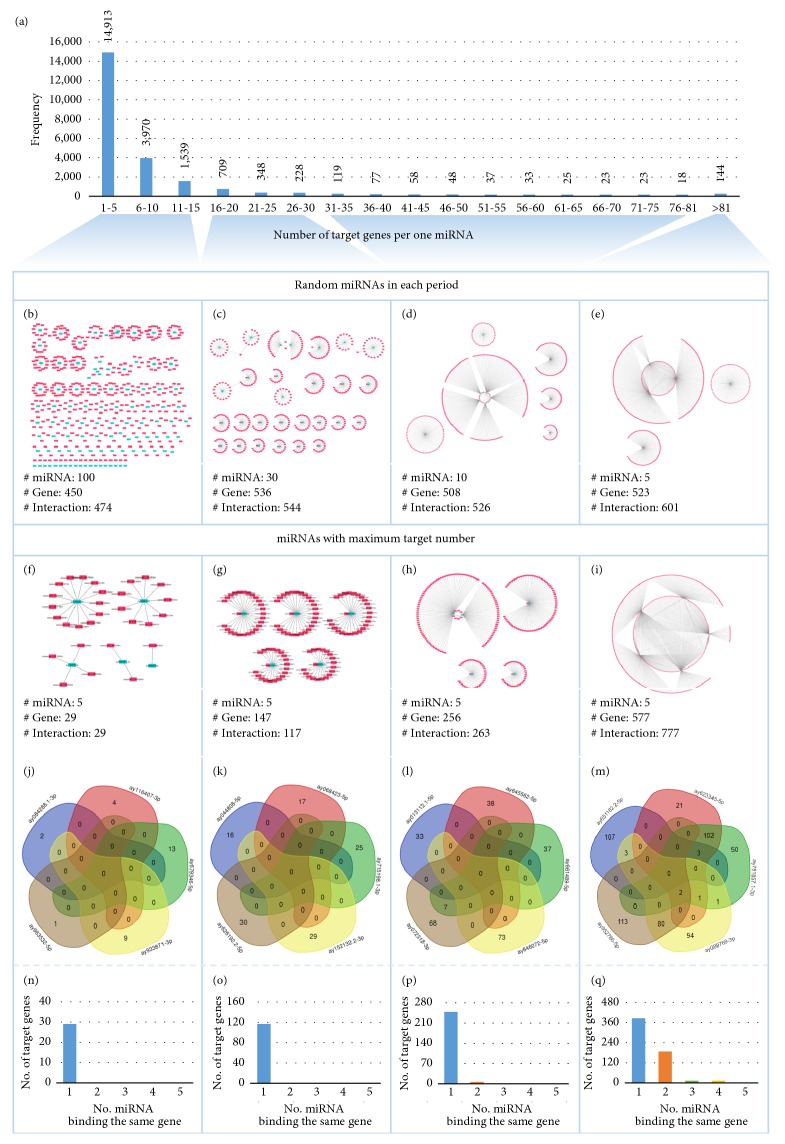
The relationship of miRNAs and target genes. (a) The distribution of a number of target genes that were bound by one miRNA. (b-e) The interactions of miRNAs (blue) and their target genes (pink), randomly selected from each group of miRNAs classed based upon the number of target genes. (f-i) The interactions of miRNAs (blue) and their target genes (pink) for the five selected miRNAs which have maximum number of target genes. (j-m) Vann diagrams represent common and unique target genes among the 5 selected miRNAs. (n-q) Bar graphs represent numbers of target genes (y-axis) that were coregulated by multiple miRNAs (x-axis).

Moreover, the relationships among miRNA-target were investigated to study their cooperation in the miRNA regulation system. The study was conducted in 4 classes of miRNA-target relationships with varied target genes to miRNA ratio, namely, (1) less than 15, (2) 16 to 30, (3) 31 to 80, and (4) greater than 81 (Figure 5(a)). The set of representative miRNA-target relationships for each group were randomly selected to preform network analysis (Figures 5(b)–5(q)). The topology of the resulting networks showed that the putative mature miRNAs associated with greater numbers of target genes tend to perform cooperative regulation among target genes (Figures 5(b)–5(e)). To demonstrate, the top 5 miRNAs ranked by the highest number of association to target genes were analyzed in-depth. Subnetworks of these 5 miRNAs (Figures 5(f)-5(i)) exhibited similar topology to their corresponding overall networks (Figures 5(b)–5(e)). The consensus target genes predicted for the putative miRNAs were illustrated in Venn diagram (Figures 5(j)–5(m)). The results suggested the coregulation of target genes by a similar miRNA and also implied the cooperation between the miRNAs in modulating the same target. The subsequent analysis in Figures 5(n)–5(q) demonstrated the corresponding figure to the network topology study. It indicated that the miRNAs targeting multiple genes are more likely to perform cooperative regulation. This finding also supported the earlier presumption of functional analysis in* LMBG* and* HMBG* groups.

### 3.7. The New Findings of Putative Mature MiRNAs and Their Relevance to the Regulation of Storage Root Development and Key Metabolism in Cassava

**Figure 6:**
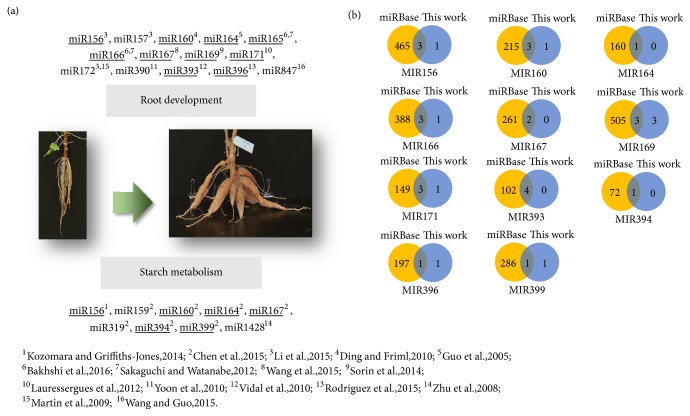
The findings of cassava miRNAs involving in regulation of root development and starch metabolism. (a) The scheme of miRNAs involved in root development and starch metabolism. The underlined miRNA families are the miRNA families found in this work, consistent with other studies; (b) Venn diagrams represented miRNA isomiRs between miRNAs from miRBase (Release21) and miRNAs in this work, where the numbers denote miRNA members in each family.

Cassava is a well-known starchy root crop. Therefore, biological processes related to growth as well as development of storage roots and also starch metabolism are always of interest. Literatures in plants have reported that miRNAs play a role in the regulation of these processes (Figure 6(a)) [20, 43–57]. miRNA165 and miRNA166 were found to be involved in the development of apical embryonic fate, either to be a shoot pole or a root pole, by regulating* HD-ZIP *III master regulator [51, 57]. miR156 and miR172 were reported to antagonistically regulate tuberization in potato [45]. miR1428 was reported to regulate starch accumulation via* SnRK1b* gene [44]. Recently, Chen et al. identified a large number of miRNAs in leaves and roots of Arg7, KU50, and W14 cassava cultivars [20]. Their functional prediction showed that miR159, miR160, miR164, miR167, miR319, miR394, and miR399 potentially bind to target genes that are involved in starch and sugar metabolisms and starch accumulation. This recent work is one of the limited studies of cassava miRNAs related to starch metabolism and accumulation. Even though many miRNAs were reported to be involved in root developmental processes, they were studied in Arabidopsis, rice, maize, legume, and soybean, but not in cassava (Figure 6).

In this work, the putative miRNAs were thoroughly predicted from cassava genome. Target gene prediction showed that most of genes in cassava genome have potential to be regulated by the putative mature miRNAs. We here proposed a range of putative miRNAs that are potentially involved in starch metabolism and storage root development. Our results correspond with findings from previous studies with great overlap as shown in the underlined marks in Figure 6(a). Furthermore, we found 9 new putative isomiRs belonging to 7 miRNA families which potentially target genes in the same functional groups (Figure 6(b) and Table S4). For example, miR156 was reported to be involved in modulation of starch content in turnip and potato [53]. The proposed putative isomiR of MIR156 (ay0909608-3p) was also predicted to target gene in starch metabolism (cassava4.1_000732m.g and cassava4.1_022381m.g in Table S4).

## 4. Discussion

In this work, putative miRNAs of cassava were thoroughly identified according to its genome sequence. The finding mature form of the putative miRNAs was not the only in-depth level of analysis for the annotation of their functions, but it also helped to certify the prediction of predicted loci in the identification step. Our results showed that putative mature miRNAs were successfully acquired for 84 percent of the predicted loci (78,022), while the remaining ~ 10 percent of them failed to form secondary structure by Mfold. An explanation for the failure could be that because loci have marginal potential for folding, structure prediction could vary with computational tools. HeteroMirPred predicts pre-miRNAs based on multiple fundamental features in addition to the folding potentials [23]. It is thus able to capture the putative miRNAs that have loose secondary structures, if their property satisfies other constituent features. This class of predicted loci might refer to an exceptional group of loose-structure miRNAs [25]. Also, it is possible that the predicted loci were identified with the marginal structure-related score. These loci could form weak secondary structures in HeteroMirPred, which are not sufficiently stable to ensure structural conformation in all computational algorithms. This is often the case for near-border predictions of classification model in bioinformatics tool [23, 58]. Mfold, which was anchored in our mature miRNA prediction process, relies on the distinct conceptual basis from RNAfold [59], an embedded tool in HeteroMirPred predictor. Mfold provides suboptimal secondary structures of the query sequences [60], whereas RNAfold gives a satisfied structural sequence [59]. The results from both tools sometimes show varying prediction. In addition, the current stringency for mature miRNA identification (Figure S1B) [34, 35] was set to ensure reliable predictions, and it might be too strict to keep the ambiguous results.

The miRNA identification, herein, focused on the entire noncoding regions of the cassava genome (approximately 98 percent of whole genome sequences). The approach allowed us to putatively identify wide miRNAs coverage, since there were no limitations on the specific conditions for gene expression and available miRNA templates as experienced in other studies [15–20, 61]. Moreover, our prediction was first screened for miRNA by multiple feature scoring and then folded to a secondary structure to capture mature miRNAs. The putative miRNAs were thus not dropped out at the early step in the identification pipeline that probably happened in previous predicted frameworks [15–19]. This method enabled us to cover extensive set of putative miRNAs beyond the structural criteria. Notwithstanding, there are some previously reported miRNAs not captured in our genome-wide scan. This suggests that there exists a computing power gap that needs improvement. The unpredicted miRNAs might be caused by the size of the sliding window employed in the input data preparation and the restriction of the selected miRNA prediction software. To improve the prediction power, overlapping sequences in the sliding window system should be extended to at least 28 nt, which is the maximum length of mature miRNAs allowed in miRBase Release21 [10]. In addition, choices of miRNA predictor that are derived by more extensive and proper training data, if available, may make the predictions more precise. Using multiple prediction tools, thereby combining the strengths of each tool could yield better results. However, time consumption should be considered for practical implementation.

To support our prediction, the miRNA candidates were verified by statistical test and their expression potentials. The analysis showed that our predictions were statistically reliable according to hypergeometric* p* value ≤ 0.05. Investigation of their expression suggests that the large number of candidates may contain species-specific miRNAs. It commonly happens in plants and is difficult to practically identify because of their low level of expression [38, 62]. More transcriptome data from diverse conditions would enable us to verify greater number of candidates and enhance the reliability of our predictions.

In addition, the genomic landscape of the predicted miRNA was studied. We proposed a different perspective of miRNA arrangement in cassava genome. It has been believed that plant miRNAs are mainly located in intergenic regions rather than genic regions [8, 36], a conclusion drawn from the number of miRNAs found, while neglecting the fact that portions of intergenic regions are generally much larger than those of genic regions. Population density analysis revealed a high density of miRNA population in genic regions rather than intergenic regions for cassava as well as for other well-studied plants (i.e., rice and soy bean). The finding led to the hypothesis that miRNAs of cassava (maybe also plants) were derived from protein-coding genes as proposed for animals, but with the distinct evolutionary paths [8, 38, 62].

Considering the conservation of the miRNA candidates, two distinguished groups can be classified. Group A class (Figure 3) of miRNAs has highly conserved nucleotide sequences as well as folded structures; therefore, they are easily identified by various identification methods. Most of the miRNAs in this group were reported to be ancient miRNAs and highly conserved in many plant species [62, 63]. They were mostly reported to be involved in biotic and abiotic stress responses, the essential regulatory processes underlying plant adaptation and overall evolutionary fitness [64]. On the other hand, the putative miRNAs of cassava in group B (Figure 3) were found in only a few plant species (Table S3). This group of miRNAs lacks structure information, which makes them more difficult to be identified using common structure-based methods. Moreover, with less conservation, few are functionally annotated such as MIR414, MIR5021, MIR845, and MIR1533 [65–67]. We hypothesized that these miRNAs that might have evolved lately, hence, are conserved within narrow range of organisms as it was reported that structures of young miRNAs evolved in specific organisms [62]. This hypothesis would be stronger if there is more evidence of such miRNA structures, but it is still practically hard.

Interestingly, MIR1533 was the first time identified in cassava with certain numbers of miRNA members. MIR1533 was found to have diverse functions in plants. The predicted targets of MIR1533 showed that it might be related to biosynthesis of plant hormones as well as starch metabolism in potato [68] and to nodule development in chickpea [67]. Moreover, MIR1533 was reported to be involved in nutrient response in sweet orange [69].

By study the role of miRNA candidates in cellular regulatory network, the different canonical role of* HMBG* and* LMBG *miRNA-target interaction groups was observed (Figures 4 and 5). The findings enabled us to perceive the contribution of miRNAs to gene regulation system. They could introduce the complicated regulation as observed in* HMBG *group and also facilitate the modulation of various function-specific genes as demonstrated in* LMBG *group. This presumption corresponds to the observed higher number of miRNA-binding sites in protein having more interaction [70] where tight regulation may be required.

Finally, the new findings of putative mature miRNAs that are related to regulation of root development and important metabolism in cassava were proposed. The results showed the extension of miRNA knowledge in cassava as our method allowed moving identification capability beyond the predefined information as well as any template-based constraints.

## 5. Conclusions

The study proposed 78,022 predicted loci containing 152,111 putative mature miRNAs. The reliability of the identified miRNAs was consolidated by the statistical test and the expression evidence. The results showed that genomic landscape of the predicted miRNAs was similar to that of well-studied plants (i.e., rice and soy bean), densely located in genic regions. Furthermore, our conservation study showed the first discovery of 10 conserved miRNAs in cassava genome from 59 putative mature miRNAs of 22 known families. Analysis of the function of the predicted miRNA candidates showed the extensive range of posttranscriptional regulation modulated by miRNAs. It was found that most genes in cassava genome were potentially bound by 151,088 putative mature miRNAs. These results implied the requirement of miRNAs in regulatory system and enabled us to envisage the landscape of miRNA-mRNA interaction at genome-scale, demonstrating the complexity of the regulation of genes by miRNAs.

## Figures and Tables

**Table 1:** The putativemiRNA sequences in cassava proposed from our genome-wide analysis.

	Intergenic			Intronic			Total		
	Number of sequences	Percentage of genome	Percentage of non-coding regions	Number of sequences	Percentage of genome	Percentage of non-coding regions	Number of sequences	Percentage of genome	Percentage of non-coding regions
Non-coding regions	56,606	91.77	93.54	142,220	6.33	6.46	198,826	98.11	100.00
120-nt input sequences	7,580,807	85.42	87.06	698,585	7.80	7.99	8,279,392	93.25	95.05
Predicted loci	70,716	2.13	2.17	7,306	0.22	0.22	78,022	2.35	2.40
Putative pre-miRNAs	58,868	0.68	0.69	6,528	0.07	0.07	65,396	0.75	0.77
Putative mature miRNA candidates									
5p	69,863	0.15	0.15	7,543	0.02	0.02	77,406	0.16	0.17
3p	67,334	0.14	0.14	7,371	0.02	0.02	74,705	0.16	0.16

**Table 2:** Statistical confidence of predicted miRNAs. Comparison between predicted loci and previously identified cassava pre-miRNAs and hypergeometric probability calculation for obtaining equal or more published pre-miRNAs in our prediction from random selection without replacement. The numbers in parenthesis represent number of miRNAs classified by family (known-family miRNAs, unknown-family miRNAs).

*Publication*	*Technique*	*Condition*	*Number of miRNAs*	*Number of miRNAs found in our prediction (X)*	*p-value∗* (Hypergeometric probability)
Patanun *et al.* 2012	Template-based method	-	151 (151,0)	15 (15,0)	4.89x10^−11^
Pérez-quintero *et al*. 2012	Deep sequencing	Bacterial infection (*Xam*)	126 (114,12)	9 (9,0)	5.27x10^−6^
Ballén-taborda *et al.* 2013	Deep sequencing	Heat and drought stress	951 (69,882)	71 (6,65)	4.28x10^−38^
Wang *et al.* 2014	Deep sequencing	Normal (fresh young leaves)	116 (105,11)	2 (2,0)	2.13x10^−1^
Xia *et al. *2014	Deep sequencing	Chilling response and stress acclimation	202 (166,36)	13 (7,6)	1.59x10^−7^

^*∗*^p (x ≥ X), where X is number of previously reported miRNAs found in our predicted loci.

## Data Availability

The catalog of putative mature miRNAs in cassava predicted in this work is available at http://bml.sbi.kmutt.ac.th/miRNAs/index.php.

## Supplementary Materials

Supplementary Materials Figure S1: the scheme of miRNAs identification work flow. (A) The framework describes miRNA identification processes in this work (gray box), which were mimicked from miRNA biogenesis in plant species (green box). Predicted loci were termed for the regions that contain at least one miRNA in their sequences. miRNA duplexes, which generally are a double stranded RNA (composing of one guide strand and one passenger strand, i.e., miRNA and miRNA*∗*, respectively), were herein referred to the stem of the stem-loop structure of pre-miRNAs. Mature miRNA candidates were then defined in this context as the single-arm sequences of the predefined miRNA duplexes. (B) The example of the miRNA duplex identification from stem-loop structure of the predicted loci (AY0000096). The miRNA duplexes (predicted according to the criteria in the gray box) were highlighted as yellow shade, where mature miRNA candidates were presumed to locate in both strands of the duplexes. Table S1: cassava RNA sequencing data from six publications supporting the existence of putative mature miRNAs. Table S2: MicroRNA densities in each organism. Table S3: cassava miRNAs that first identify in this work (black sequences) and their conserved miRNA of organisms in miRBase Release21 (blue sequences). Table S4: the functions of new isomiRs predicted by psRNATarget.
